# Preoperative elevated FDP may predict severe intraoperative hypotension after dural opening during decompressive craniectomy of traumatic brain injury

**DOI:** 10.1186/s40981-018-0146-5

**Published:** 2018-01-16

**Authors:** Kei Kamiutsuri, Naoki Tominaga, Shunji Kobayashi

**Affiliations:** 1 Department of Anesthesiology, Rinku General Medical Center, Izumisano, Japan; 2 Department of Cardiovascular Internal Medicine, Shin Komonji Hospital, Kitakyushu, Japan

**Keywords:** Decompressive craniectomy, Coagulation disorder, Fibrinogen degradation product (FDP), Intraoperative hypotension, Dural opening

## Abstract

**Purpose:**

Coagulation disorder and intraoperative hypotension are representative complications of traumatic brain injury which cause worse perioperative outcome. The aim of this study was to survey the relation of coagulation disorder and intraoperative hypotension (IH) during decompressive craniectomy.

**Method:**

Patients who underwent emergency decompressive craniectomy due to traumatic brain injury were retrospectively surveyed. The relation between preoperative coagulation date and intraoperative hypotension (systolic blood pressure < 60 mmHg after dural opening) was analyzed.

**Results:**

Of 41 patients screened, 12 patients (27.9%) developed IH. Fibrinogen degradation products (314 vs 64.4 μg/mL; *p* = 0.01) were significantly higher in the IH group. In contrast, fibrinogen (181 vs 239 mg/dL; *p* = 0.01) was significantly lower in the IH group. Reduction rate of sBRP before and after dural opening (%) was higher in IH group than in non-IH group (49.1 vs 27.6%: *p* = 0.001).

**Conclusions:**

Preoperative elevated FDP may predict IH after dural opening during traumatic decompressive craniectomy.

## Introduction

Traumatic brain injury (TBI) is one of the most critical and major concerned diseases worldwide [1, 2]. As a major strategy of TBI that requires emergent reduction of the intracranial pressure, decompressive craniectomy (DC) is established, a surgical method to decrease intracranial hypertension and to ensure adequate cerebral perfusion pressure [3, 4]. However, considering anesthetic management during DC, intractable hypotension which is unresponsiveness to several vasopressors often occurs after dural opening and it sometimes develops cardiac arrest [5]. Although these circulatory failures during DC often occur, the contributing factors for intraoperative circulatory failure are not sufficiently researched and discussed.

Coagulation disorder triggered by TBI is also a major complication of TBI [6], which often induces intraoperative hemostatic disorder, postoperative cerebral edema, or postoperative hemorrhage. Furthermore, coagulation disorder triggered by TBI is reportedly associated with intraoperative severe brain swelling [7], severity of TBI [8], and poor outcome [9–11].

We experienced several cases complicated with severe intraoperative hypotension after dural opening during emergent DC of which preoperative coagulation laboratory values were remarkably outside of the normal range. Although intraoperative circulatory failure and coagulation disorder are representative complications for TBI surgery, the relation between intraoperative circulatory failure and coagulation disorder has not been discussed. From these clinical experiences, we hypothesized that coagulation disorder triggered by TBI may be related to intraoperative hemodynamic instability in patient with TBI and that preoperative coagulation disorder may become a predictive factor of IH following dural opening during emergent DC. The aim of our study was to investigate the correlation between coagulation disorder and IH after dural opening during emergent DC and to identify coagulation disorder as a risk factor of IH.

## Materials and methods

We adhered to the Declaration of Helsinki. Following approval by the institutional review board of Osaka City General Hospital, we conducted a retrospective study in a cohort of patients aged above 18 years who were transferred directly from the emergency room (ER) to the operating room (OR) for emergent craniotomy between May 2009 and November 2014 at the Osaka City General Hospital. We surveyed all cases of emergent DC required dural opening due to traumatic acute subdural hematoma or intracerebral hemorrhage which were performed within 12 h from injury. We excluded patients with medical history of ischemic or cardiovascular disease, chronic renal disease, chronic respiratory disease, and cerebrovascular disorder and also excluded those with insufficient medical charts, unknown mechanism of injury, and insufficient preoperative screening date. We also excluded craniotomy not required dural opening such as epidural hematoma, burr hole evacuation procedures, and repeated craniotomy from this study.

The following data were obtained from the medical records and emergency department records: age, gender, hemodynamic data, body weight, blood pressure and heart rate on admission at ER, Glasgow Coma Scale (GCS) score, neurologic symptom (pupil asymmetry, loss of light reflex), body temperature on arrival at ER, mechanism of injury (traffic injury or not), Injury Severity Score [12], type of intracranial injury (acute subdural hematoma, intracerebral hemorrhage, acute subdural hematoma) by computed tomography (CT), laboratory values (abbreviation, normal range) first collected on arrival at ER; white blood cell (WBC 3.3~8.6 × 10^3^/μL), hemoglobin (Hb 11.6~16.8 g/dL), platelet count (PC 15.8~34.8 × 10^4^/μL), lactate (Lac 4.2~17.0 mg/dL), C-reactive protein (CRP < 0.14 mg/dL), fibrinogen (Fbg 150~400 mg/dL), fibrinogen degradation products (FDP < 5 μg/mL), prothrombin time international normalized ratio (PT-INR 0.85~1.15), activated partial thromboplastin time (aPTT 24.3~36.0 s), treatment in ER; mannitol use, vasodilator use (nicardipine, nitroglycerin), vasopressor (ephedrine, phenylephrine, norepinephrine, epinephrine) use, blood transfusion, endotracheal intubation on ER, and time from estimated injury to operation.

The following intraoperative variables were also obtained from electronic anesthetic record: intraoperative bleeding (ml/kg), vasopressor use before dural opening (ephedrine, phenylephrine, epinephrine, norepinephrine), mannitol use before dural opening, operation time, intraoperative use of inhalation anesthetic, use of remifentanil before dural opening, blood pressure and heart rate on arrival at OR, and before and after dural opening.

Our study was a retrospective survey; therefore, there was no standardized protocol for the anesthetic management of the patients undergoing DC during the study period, and the anesthetic regime was determined by the anesthesiologist in charge. General anesthesia was maintained by intravenous propofol or by inhalation of sevoflurane combined with continuous remifentanil or by intermittent boluses of fentanyl and rocuronium. The choice of a vasopressor (ephedrine, phenylephrine, norepinephrine, or epinephrine) depended on the anesthesiologist in charge. Mannitol was used to induce brain relaxation, if required by the neurosurgeon. In addition to standard monitors, arterial catheters were used for continuous blood pressure monitoring and for sampling blood gas. All patients were transferred to the intensive care unit of the emergency and critical care center after the surgery.

### Definition of IH

We defined IH as systolic blood pressure (sBP) < 60 mmHg occurring within 15 min of dural opening in this study. Patients with sBP < 60 mmHg before dural opening were excluded from this study. Blood pressure was measured by using arterial blood pressure monitoring on the radial artery during operation and recorded in the electronic anesthetic record.

### Statistical analysis

Statistical analysis was performed with Bell Curve for Excel (Social Survey Research Information Co., Ltd. Japan). All data are presented as medians (interquartile range, 25th–75th percentile) or number with percentages. Parameters in patients with and without IH were compared using Mann–Whitney *U* tests, Fisher’s exact tests, and chi-square tests as appropriate. Odds ratios are presented with 95% confidence intervals (CI). *p* values less than 0.05 were considered statistically significant.

## Results

Seventy-nine patients underwent emergent DC during this period, and of these, 41 patients who met the criteria for the study were subsequently included in this study. Of these 41 patients, 12 patients (27.9%) developed IH after dural opening during emergent DC. Hemodynamic variables (sBP, dBP, HR) from arrival at ER to after dural opening during DC were shown in Table 1. sBP, dBP, and HR were not significantly different in both groups from arrival at ER to before dural opening; however, sBP and dBP after dural opening were significantly lower in IH group (54 vs 83 mmHg; *p* = 0.008, 34 vs 44 mmHg; *p* = 0.002). Reduction rate of sBP before and after dural opening was higher in IH group than in non-IH group (49.1 vs 27.6%; *p* = 0.001); however, reduction rate of HR before and after dural opening was not significantly different in both groups (3.91 vs − 1.96%; *p* = 0.61).

**Table 1:** Hemodynamic date from arrival at ER to after dural opening

	IH *n* = 12 (IQR)	Non-IH *n* = 29 (IQR)	*p* value
sBP on arrival at ER (mmHg)	156 (109–192)	130 (120–151)	0.3
dBP on arrival at ER (mmHg)	98 (75–120)	80 (63–93)	0.05
HR on arrival at ER (/min)	80 (80–109)	80 (71–106)	0.8
sBP on arrival at OR (mmHg)	111 (99–137)	131 (123–146)	0.08
dBP on arrival at OR (mmHg)	58 (50–67)	66 (60–81)	0.06
HR on arrival at OR (/min)	103 (80–109)	92 (78–107)	0.51
sBP before opening dura (mmHg)	97 (88–118)	110 (100–128)	0.09
dBP before opening dura (mmHg)	56 (52–60)	57 (52–72)	0.46
HR before opening dura (/min)	108 (98–120)	90 (76–104)	0.07
sBP after opening dura (mmHg)	54 (48–58)	83 (71–86)	0.008
dBP after opening dura (mmHg)	34 (29–37)	44 (40–50)	0.002
HR after opening dura (/min)	103 (93–115)	90 (76–100)	0.05
Reduction rate of sBRP before and after dural opening (%)	49.1 (39.2–54.6)	27.6 (21.1–35.1)	0.001
Reduction rate of HR before and after dural opening (%)	3.91 (− 12.5 to 9.99)	− 1.961 (− 6.667 to 3.488)	0.61

*sBP* systolic blood pressure, *dBP* diastolic blood pressure, *HR* heart rate, *ER* emergency room, *OR* operation room

Patient characteristics were shown in Table 2, and demographic date, laboratory variables, radiographic findings, and operation date were shown in Table 3. Total amount of intraoperative bleeding (25.3 vs 8.6 ml/kg; *p* = 0.005) and FDP (314 vs 64.4 μg/mL; *p* = 0.01) was significantly higher in IH group. In contrast, Fbg (181 vs 239 mg/dL; *p* = 0.01) was significantly lower in IH group.

**Table 2:** Patient characteristics

Gender (male)	5 (38.4)	20 (68.9)	0.32 (0.07–1.29)	0.1
Multiple trauma	6 (50)	11 (37.9)	1.63 (0.42–6.36)	0.47
Traffic trauma	9 (75)	13 (44.8)	0.62 (0.18–2.14)	0.45
Loss of light reflex	7 (58.3)	20 (68.9)	0.63 (0.15–2.53)	0.51
Pupil asymmetry	3 (25)	11 (37.9)	0.54 (0.12–2.46)	0.43
ASDH	7 (58.3)	15 (51.7)	1.4 (0.35–5.48)	0.62
ICH	1 (8.3)	8 27.5)	0.22 (0.25–2.06)	0.18
ASDH and ICH	4 (33.3)	6 (20)	1.83 (0.41–8.23)	0.42
Endotracheal intubation on ER	8 (66.6)	21 (72.4)	0.76 (0.17–3.24)	0.71
Mannitol use on ER	8 (66.6)	16 (55.1)	1.62 (0.39–6.62)	0.49
Vasodilator use on ER	4 (33.3)	3 (10.3)	2.08 (0.38–11.1)	0.39
Vasopressor use on ER	3 (25)	3 (10.3)	2.83 (0.49–16.9)	0.24
Blood transfusion on ER	3 (25)	3 (10.3)	2.83 (0.49–16.9)	0.24
Mannitol use before opening dura	12 (100)	15 (51.7)	1.3 (0.33–5.08)	0.69
Intraoperative use of inhalational anesthetic	10 (83.3)	25 (86.2)	0.8 (0.12–5.08)	0.81

*ASDH* acute subdural hematoma, *ICH* intracerebral hemorrhage, *ER* emergency room

**Table 3:** Demographic date, laboratory variables, radiographic findings, and operation date of patients with or without IH

	IH *n* = 12 (IQR)	Non-IH *n* = 29 (IQR)	*p* value
Age (year)	64 (39–68)	52 (30–63)	0.44
Body weight (kg)	55 (51.5–62)	63 (55–68)	0.25
GCS	9(7–13)	8 (6–10)	0.18
ISS	21 (16–29)	17 (17–25)	0.98
WBC (10^3^/μL)	10.3 (9.2–15.3)	10.4 (8.8–13.2)	1.00
Hb (g/dL)	13.3 (12.3–13.8)	13.6 (11.425–14)	0.66
CRP (mg/dL)	0.05 (0.03–0.3)	0.05 (0.03–0.21)	0.47
Lac (mg/dL)	25 (19–38)	25 (15.25–39)	0.91
PC (× 10^3^/μL)	180 (134–247)	225 (187–252)	0.14
aPTT	30.0 (29.3–33.9)	28.0 (25.8–31.1)	0.03
PT-INR	1.06 (1.0.2–1.14)	1.0 (0.97–1.06)	0.01
FDP (μg/mL)	314 (175–439)	64.4 (39.9–98.5)	0.01
Fbg (mg/dL)	181 (134–238)	239 (203–271)	0.02
CT midline shift (mm)	10 (8–14)	10 (8–12)	1.0
Body temperature on arrival at ER (°C)	35.3 (35.2–35.9)	36.2 (35.3–36.4)	0.22
Total amount of fentanyl before opening dura (μg/kg)	2.47 (1.25–3.3)	3.17 (2.38–4.38)	0.07
Time from injury to operation (h)	3 (2.7–3.2)	5 (3–9)	0.09
Operation time (h)	2.93 (2.01–3.85)	3.21 (2.65–3.38)	0.55
Intraoperative bleeding (mL/kg)	25.3 (19.9–41.1)	8.6 (5.2–14.4)	0.005
Anesthetic time (h)	3.4 (2.37–4.64)	3.86 (3.14–4.41)	0.26
Operation time (h)	2.76 (1.79–3.87)	3.22 (2.65–3.58)	0.28

*GCS* Glasgow Coma Scale, *ISS* Injury Severity Score, *WBC* white blood cell, *Hb* hemoglobin, *PC* platelet count, *CRP* C-reactive protein, *Lac* lactate, *PC* platelet count, *aPTT* activated partial thromboplastin time, *PT-INR* prothrombin time international normalized ratio, *FDP* fibrinogen degradation products, *CT* computed tomography

## Discussion

The main finding of this study was that coagulation disorder was associated to intraoperative circulatory failure followed by dural opening during DC. From previous reports, low GCS [13, 14], tachycardia, hypertension before emergency craniotomy [13], delayed surgery [13], absence of the mesencephalic cistern on CT scan, bilaterally dilated pupils [14], the presence of multiple CT lesions, subdural hematoma, maximum thickness of CT lesion, and longer duration of anesthesia [15] were the risk factors of IH in adult traumatic brain injury patients. Although these physical findings or radiographic finding by CT scan were reported as risk factors of IH, quantitative parameters such as laboratory values have not been reported as risk factors of IH. Abnormal laboratory values of coagulation disorder may be indicators to predict intraoperative circulatory failure, as well as physical or radiographic finding, if more evidence is established.

Coagulation disorder triggered by TBI is related to unfavorable neurological outcome [6], higher mortality [10], or severity of TBI [8]; however, it does not always provide effective information about intraoperative hemodynamics of DC. Although there was a significant difference in aPTT and PT-INR statistically in this study, the difference was very small and within normal range, which may have low clinical importance. Decreased Fbg of IH group also has less clinical value because Fbg of both groups were also within normal range and decreased Fbg is popular for clinical manifestation by multiple trauma and this result (Fbg 181 mg/dL) may not be appropriate to define severe criteria for multiple trauma. In contrast to aPTT, PT, and Fbg, FDP of both groups exceeded a normal range (< 5 μg/mL) and FDP of IH group (314 μg/mL) was significantly higher than that of non-IH group (64.4 μg/mL). This characteristic abnormality of laboratory value shown by FDP was not observed in aPTT, PT, and Fbg; therefore, elevated FDP may have a more diagnostic value as a predictive marker than other aPTT, PT, and Fbg. Although elevated FDP is reported as a predictive factor of deterioration requiring surgery of TBI [16] or as high mortality during ICU stay [17], clinical utility of FDP is not still established in the intraoperative management of TBI. Our result may be one of the beneficial findings to predict intraoperative circulatory failure of DC.

Both sBP and dBP on arrival at ER and OR and before dural opening of each group were not significantly different, and HR of both group did not almost change before and after dural opening. Since total amount of intraoperative bleeding was higher in IH group, we were not always able to exclude the influence of intraoperative bleeding. However, considering that the total amount of intraoperative bleeding was not counted just after dural opening but at the end of operation and preoperative hemoglobin concentration and vasopressor use on ER, blood transfusion on ER was not different in each group, the influence of total amount of intraoperative bleeding may have a low impact on intraoperative circulatory failure followed by dural opening during DC. Considering these factors, this unique hemodynamical change indicates that blood pressure reduction after dural opening was not mainly affected by hypovolemia due to bleeding or dehydration. One of the mechanisms causing intraoperative hemodynamic instability after dural opening is a reduced vascular resistance by a sudden decrease in sympathetic tone following the relief of increased intracranial pressure by dural opening [14, 18, 19]. However, it is practically difficult to predict abnormal increase of sympathetic tone by measuring catecholamine level on admission, since it is almost unable to know catecholamine level in actual clinical settings. Therefore, an alternative approach to predict abnormal increase of sympathetic tone that indicates high catecholamine level is still a focus of interest in the management of TBI. From Di Battista’s reports, sympathoadrenal activation measured by catecholamine levels was associated with acute traumatic coagulopathy [20]. Considering the relation of TBI and elevated catecholamines or the relation of acute traumatic coagulopathy and catecholamine levels, preoperative coagulation disorder triggered by TBI may reflect increased abnormal sympathetic tone which is not detectable from preoperative radiographic findings by using CT or by preoperative physical findings. Our result confirms this hypothesis and may reveal the detailed mechanism of intraoperative hemodynamic instability during DC.

The definition of IH that we used in this study, systolic blood pressure < 60 mmHg, may be controversial in comparison with definitions of IH ever reported. Several definitions of IH, a systolic blood pressure below 80 mmHg, a decrease of systolic blood pressure more than 20% below baseline, or combinations of absolute and relative systolic blood pressure thresholds SBP < 100 mmHg or 30% decrease from baseline, were often used [21]. sBP < 90 mmHg [13, 15] or reduction of mean arterial pressure > 20% of baseline values (5 min before opening the dura) [14] were used as the definition of IH in TBI surgery. However, in our study, sBP after dural opening of non-IH group (83 mmHg) was lower than 90 mmHg and we did not evaluate circulatory dynamics by mean arterial pressure. Therefore, we were obliged to use an alternative definition of IH. Considering that the definition of IH is not always established and several blood pressure measurements were individually used as IH in many studies [21], sBP < 60 mmHg may be a permissible range to define IH in this study.

### Limitations

There were some limitations in this study. First, this was a retrospective study conducted at a single medical center. The total number of case was not sufficient, and there was no standardized protocol for preoperative and intraoperative anesthesia management. Second, our study involved not only isolated TBI patients, but also TBI patients with multiple trauma. To examine the relation of preoperative coagulation disorder and intraoperative circulatory failure of TBI surgery without influence of other sites of trauma, the study to collect only isolated TBI patients may be preferable. Third, detailed perioperative cardiovascular examinations including transesophageal echocardiography, pulmonary artery catheter, or arterial pulse contour-based cardiac output device were not always performed in all patients and a differential diagnosis of intraoperative hypotension was insufficient. A prospective, large-scale, randomized study with enough sample size and detailed hemodynamic measurement is needed to confirm the relation of preoperative coagulation disorder and intraoperative circulatory failure.

## Conclusion

Elevated FDP was related to IH after dural opening during traumatic decompressive craniectomy, and elevated FDP may become one of the predictive factors for IH during traumatic decompressive craniectomy.
